# Combining multivariate analysis and monosaccharide composition modeling to identify plant cell wall variations by Fourier Transform Near Infrared spectroscopy

**DOI:** 10.1186/1746-4811-7-26

**Published:** 2011-08-18

**Authors:** Andreia M Smith-Moritz, Mawsheng Chern, Jeemeng Lao, Wing Hoi Sze-To, Joshua L Heazlewood, Pamela C Ronald, Miguel E Vega-Sánchez

**Affiliations:** 1 Joint BioEnergy Institute, Lawrence Berkeley National Laboratory, One Cyclotron Road MS 978-4101, Berkeley, CA 94720, USA; 2 Physical Biosciences Division, Lawrence Berkeley National Laboratory, One Cyclotron Road MS 978-4101, Berkeley, CA 94720, USA; 3 Department of Plant Pathology, University of California, One Shields Ave., Davis, CA 95616

**Keywords:** near infrared spectroscopy, cell wall, hemicellulose, multivariate analysis, mutant screen, pls modeling

## Abstract

We outline a high throughput procedure that improves outlier detection in cell wall screens using FT-NIR spectroscopy of plant leaves. The improvement relies on generating a calibration set from a subset of a mutant population by taking advantage of the Mahalanobis distance outlier scheme to construct a monosaccharide range predictive model using PLS regression. This model was then used to identify specific monosaccharide outliers from the mutant population.

## Background

Plant cell walls are a complex mixture of polysaccharides, proteins and the phenolic polymer lignin that have been recently targeted as possible sources of fermentable sugars for the production of biofuels and other bio-materials [1]. The development of a lignocellulose biomass-based biofuels industry is partly dependent on genetic engineering and breeding of the next generation of crops containing, among other traits, easily extractable cell wall sugars. Thus, a better understanding of how plants synthesize, deposit and modify their cell walls is necessary for the selection of traits important for biofuel crop improvement [2].

The identification of plants with altered cell wall composition or structure can prove useful in the discovery of novel genes involved in the biosynthesis and modification of the cell wall. Such plants can be isolated using genome-wide association mapping of diverse populations or can be isolated from forward genetic screens, where a subset sample population with the desired traits is selected from a large pool of mutagenized individuals. However, the identification of these select samples requires a well-constructed screening process that is both robust and, due to the large sample population, high-throughput. Several successful plant cell wall mutant screens have been described over the years that make use of different screening methodologies. These include: acid hydrolysis and monosaccharide composition using gas-liquid chromatography [3], microscopic observation of xylem stem sections [4,5], seedling growth on medium containing cell wall hydrolyzing enzymes [6] and Fourier-Transform Infrared (FT-IR) microspectroscopy [7,8]. Most of these approaches either required at least some kind of sample processing or were not amenable to high-throughput screening, especially when dealing with, in some cases, thousands of mutagenized plant samples. In addition, most of these screens have been performed on the model species *Arabidopsis thaliana*, a dicot, which is known to have a different cell wall type than grasses [9].

Recently various infrared spectroscopy techniques such as Fourier Transform Mid-Infrared (FT-MIR) have been used to characterize plant cell wall model compounds and mutants [7,8,10-16]. Due to the chemical specificity of this infrared region (400 to 4000 cm^-1^), one can directly identify certain peaks related to cell wall components. However, the use of FT-MIR in these studies involved careful plant cell wall extraction and/or probing of individual plant cells with a FT-MIR microscopy objective. Though very effective and informative, the use of FT-MIR as a high throughput cell wall screening technique for a large population is not practical due to the need for meticulous sample handling.

Significantly, another region of the infrared spectrum, the near-infrared (NIR), has shown promise in the classification and characterization of plant material in a more rapid manner. In contrast to MIR, the NIR region (12000 to 4000 cm^-1^) does not reveal discrete signature peaks, but it excites several harmonic overtones of methyl, aromatic CH-OH, with minor features in methoxy and carbonyl CH bonds, generating spectra that have no easily distinguishing chemical features [17]. However, with the help of multivariate analysis to deconvolve the spectrum, FT-NIR has been successfully applied to rapidly quantify and classify numerous known components in complex mixtures [18-20]. In this manner, cell wall components such as carbohydrates, ash content, and lignin have been successfully modeled and cross-validated from a defined plant set of various tissue types [21-27]. In order to correlate NIR spectra to chemical features and eventually quantify individual components in a mixture, a robust training set containing NIR spectra of a range of known concentrations is required. Using Partial Least Squares (PLS) regression, a model can then be developed to determine the concentration of these components in unknown mixtures, within the same range, by using NIR spectra alone [28]. Successful applications of FT-NIR techniques for fast chemical characterization involve acquiring accurate sample spectra, applying robust chemometric/multivariate analysis for spectra processing and obtaining reliable calibration sets for modeling. Recently, FT-NIR and linear discriminate analysis (Mahalanobis distance) were used to screen a mutant maize population to identify putative mutants [29,30]. In this study, approximately 1.8% of the samples were identified as putative mutants and 6 of these (17% validation rate) were confirmed by pyrolysis-molecular beam mass spectrometry. While highlighting the effectiveness of FT-NIR analysis in the discrimination of plant samples, the procedures outlined in these publications [30,31] were limited in application details and no chemometric analysis (e.g. PLS modeling) were performed.

The non-destructive, fast and quantitative nature of NIR spectroscopy makes it a very attractive option to use for screening samples in large plant populations. This study outlines a detailed process for the application of fast scanning of intact plant leaves by NIR spectroscopy followed by an outlier detection scheme combining linear discriminate analysis and PLS modeling. The approach was validated on known cell wall mutants of rice and *Arabidopsis *and then applied to a rice mutant collection consisting of thousands of uncharacterized samples. The technique involves first nonspecific outlier detection using Mahalanobis distance analysis of NIR spectra followed by the development of a predictive model that could be readily implemented for a variety of analyses and applied to any collection of plant mutants or variants. We show that this approach significantly improves outlier detection over the Mahalanobis distance alone, as well as allowing the identification of specific cell wall variants in the mutant population.

## Results

### FT-NIR analysis of Arabidopsis cell wall mutants

**Figure 1:**
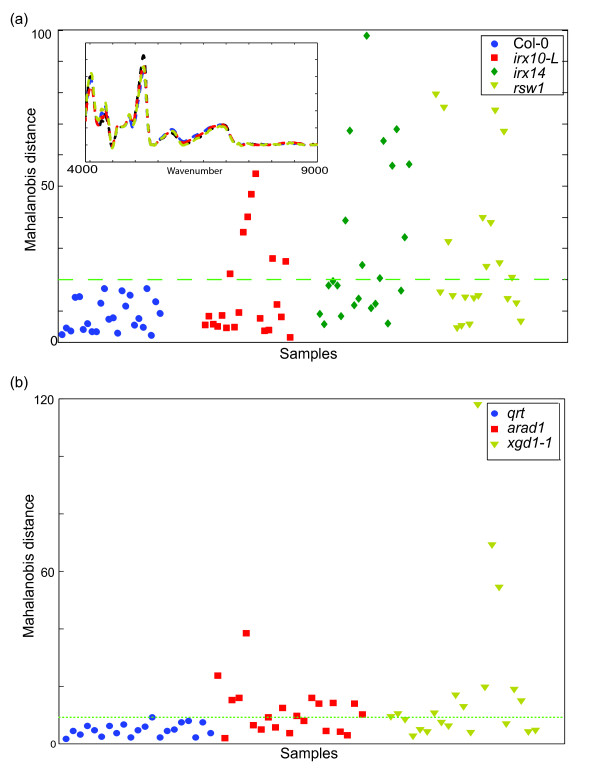
**FT-NIR and multivariate analysis of *Arabidopsis *rosettes**. **(a) **Mahalanobis distance calculated from FT-NIR spectra of the cell wall mutant plants *irx10-L, irx14, rsw1 *with spectra from wildtype (Col-0) as the reference. Inset is an example of area-normalized and baseline corrected FT-NIR spectra for cell wall mutants. **(b) **Mahalanobis distance calculated from FT-NIR spectra of the cell wall mutant plants *arad1 and xgd1-1 *with the *qrt *background plants as the reference.

In order to evaluate the effectiveness of FT-NIR in clustering different plant populations without the need of cell wall extraction or processing, various characterized *Arabidopsis *cell wall mutants were analyzed (Table 1). Whole *Arabidopsis *rosettes were dried and used for subsequent analysis by FT-NIR. A portion of each whole rosette was placed directly on the 1 cm diameter sample window of a FT-NIR MPA and three separate measurements were taken at random locations on the rosette, including both the adaxial and abaxial sides. A total of six to eight individual rosettes were measured in this way for each plant line. A representation of pre-processed and area-normalized NIR spectra from cell wall mutants in the Columbia (Col-0) genetic background is outlined in Figure 1a (inset). Due to a lack of chemical specificity inherent to FT-NIR, there were no obvious differences that could be visually discerned between the spectra of the cell wall mutants when compared to wildtype (WT) rosettes, and thus required additional data processing.

**Table 1:** *Arabidopsis thaliana *mutants used in this study.

Mutant	Phenotype	Mutated gene	Background	Reference
*irx10-L*	non-discernible	At5g61840	Col-0	[35,36]
*irx14*	irregular xylem, xylan-deficiency	At4g36890	Col-0	[33]
*rsw1*	primary cell wall cellulose deficiency	At4g32410 (*AtCesA1*)	Col-0	[34]
*arad1*	pectic arabinan deficiency	At2g35100	*qrt*	[32]
*xgd1-1*	xylogalacturonan deficiency	At5g33290	*qrt*	[31]

Principal component analysis (PCA) has been widely demonstrated to be an effective data compression technique where a new basis set (principal components axes) representing the maximum variance across the whole sample set is calculated. For data compression, PCA was performed on pre-processed and area-normalized NIR spectra of *Arabidopsis *WT (Col-0) and cell wall mutants *irregular xylem 10-Like *(*irx10-L*), *irregular xylem 14 *(*irx14*) and *radially swollen 1 *(*rsw1*). This was followed by calculation of the Mahalanobis distance, a linear discriminate analysis (LDA) technique, to identify outliers when compared to the WT background Col-0. The 10 principal component scores (accounting for 90% of the variability in the entire population) for the Col-0 rosettes were used as the reference set to calculate a single Mahalanobis distance for cell wall mutant rosettes (Figure 1a). An analysis of the cell wall mutants *arabinan deficient 1 *(*arad1*) and *xylogalacturonan deficient 1 *(*xgd1-1*) in the *quartet *(*qrt*) background was also undertaken using the same process outlined above (Figure 1b). In both these examples, any data point greater than the largest Mahalanobis distance for the corresponding WT background was considered outside the biological variance and therefore identified as an outlier. This process demonstrates that, although we had randomly scanned whole rosettes comprising of various leaf developmental stages, half of the measurements from known cell wall mutants were identified as outliers. We surmised that even though only a portion of the mutants were identified, it was possible to use NIR and subsequent multivariate data analysis of unprocessed plant material as a first pass outlier detector scheme in a rapid manner.

In order to correlate the FT-NIR analysis with actual changes in the cell wall, we performed monosaccharide composition analysis of extracted cell wall material following trifluoroacetic acid (TFA) hydrolysis and high performance anion exchange chromatography (HPAEC). The TFA treatment mainly hydrolyses the matrix polysaccharides in the plant cell wall (pectin and hemicelluloses). We must point out here that the HPAEC protocol that we have used fails to resolve xylose and mannose efficiently. For *Arabidopsis*, mannose represents between 5-10% of the primary cell wall [10] and thus we have labeled it as the mannose/xylose value in the figure. When we refer to mol_% _values, these only represent the TFA hydrolysate component of the cell wall (hemicelluloses and pectin fractions).

Cell wall mutants *irx14 *(Additional file 1: Figure S1a), *arad1 *and *xgd1-1 *(Additional file 1: Figure S1b) clearly showed a decrease in xylose, arabinose and xylose, respectively, as has been reported previously [31-33]. Although not showing significant differences in matrix polysaccharide sugar composition, cell wall mutants *rsw1 *which is impaired in cellulose accumulation [34] and *irx10-L *[35,36] can also be identified as outliers using FT-NIR and multivariate analysis (compare Figures 1 and Additional file 1: Figure S1). FT-NIR analysis coupled to Mahalanobis distance analysis thus shows that it can discern more than just differences in cell wall composition.

### FT-NIR analysis of rice mutants

**Figure 2:**
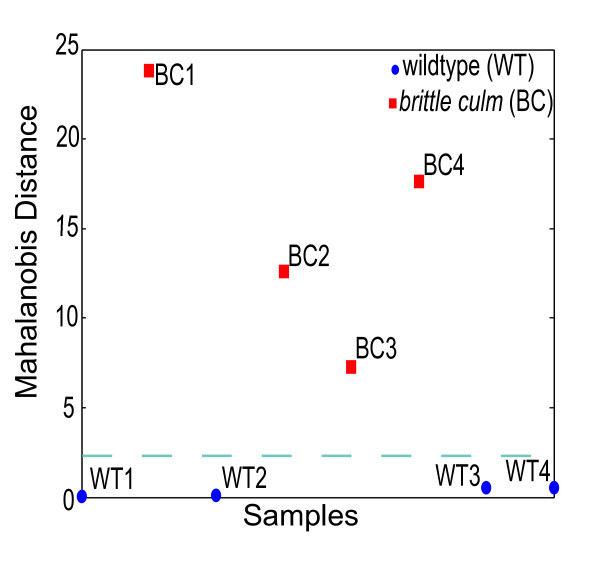
**FT-NIR and multivariate analysis of rice**. Mahalanobis distance analysis of the rice *brittle culm *(*bc*) mutant and wildtype (WT, cultivar Nipponbare). The distance was based on the first principal component score of the FT-NIR spectra with WT1 and WT2 used as the reference set against all BC samples with WT3 and WT4 employed as the test set.

In order to test the viability of reducing the number of measurement scans to a single scan of a single tissue type and developmental stage, a known rice cell wall mutant and its corresponding WT were analyzed. A rice mutant line containing a transposon insertion in the *CELLULOSE SYNTHASE A7 *(*OsCESA7*) gene has previously been shown to cause the brittle culm phenotype due to a dramatic reduction in secondary cell wall cellulose deposition [37]. We performed FT-NIR scanning of 3-week-old leaves from four WT rice plants (cultivar Nipponbare) and the *brittle culm *mutant *Oscesa7*. The WT samples were randomly assigned into two groups; one was used as a reference set and the other as a validation set. After preprocessing, area normalization and taking the PCA of the spectra, WT1 and WT2 were employed as the reference set to determine the Mahalanobis distance for the other two WT samples (WT3 and WT4) and all the *brittle culm *samples (BC1-4) samples (Figure 2). Due to the fact that only two samples were used as controls, a single principal component score was used in the calculation, accounting for 60% of the variability. The largest Mahalanobis distances corresponded to the four *brittle culm *mutant samples while the validating WT samples clustered with the reference set (Figure 2). This demonstrates that the first principal component score based on a single FT-NIR scan has the ability to distinguish differences between biological replicates of rice mutants and WT in whole rice leaves, and that the technique could be used to analyze samples in a high-throughput manner (1 scan per sample).

### Utilizing the Mahalanobis distance to determine outliers in a rice mutant population

**Figure 3:**
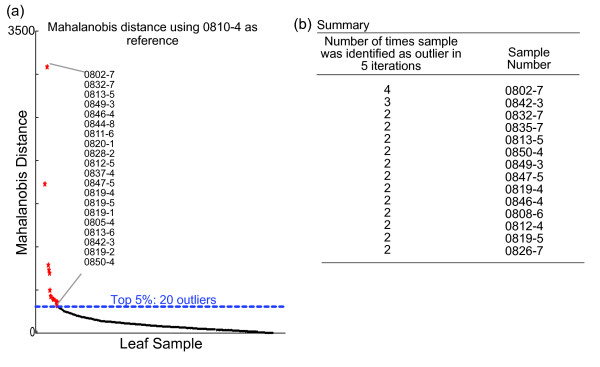
**Example of screening process using Mahalanobis distance**. Mahalanobis distance outlier screening of a single batch (370 leaf samples) of the rice mutant population. **(a) **A single iteration of the Mahalanobis distance analysis for batch 0800-0850 based on one randomly chosen reference sample (sample 0810-4). The rest of the samples (*x*-axis) were sorted in descending order of their distance (*y*-axis) compared to the reference sample. **(b) **Summary of outliers based on five iterations of Mahalanobis distance calculations using randomly selected reference sets from the same batch.

We were interested in assessing the feasibility of this strategy to identify outliers in a rice mutant population consisting of thousands of unknown samples. We used a mutant population that was generated by fast neutron bombardment of the rice line Kitaake-Ubi-Xa21 (Chern and Ronald, unpublished). We devised a pilot study consisting on the analysis of 3 week-old leaf samples collected from 550 mutant lines (segregating, M2 generation). Because the plants were grown in a greenhouse in batches of 50 lines, our experimental unit was defined as a batch of 50 lines, with each line consisting of 8 independent leaf samples (approximately 400 leaf samples per batch). This was done to control for variations in environmental conditions between the 11 pilot batches grown at different times in the greenhouse. In addition, batch specific references were required to account for biological variability. A single leaf sample was randomly chosen from the batch and was scanned five times at various locations on the leaf. This sample and its corresponding replicate scans were then designated as one of the NIR reference sets specific for that batch. We reasoned that it was highly unlikely that a randomly selected leaf in this population was a cell wall mutant, but would be a most probable representative of WT lines. Each of the leaves in the entire batch were then placed on the FT-NIR sample window and scanned once. Mahalanobis distance was subsequently determined for all samples based on the first 4 principal component scores of a FT-NIR leaf spectrum and the defined NIR reference set. An example of a Mahalanobis distance result is shown in Figure 3a, where all samples in a batch are listed in descending Mahalanobis distance from the NIR reference set (0810-4). The top 5% (~ 20 samples) representing the largest Mahalanobis distances from the reference set were identified and recorded. This process was repeated four more times with other randomly selected samples from the batch to serve as reference sets for analysis. The leaf samples that appeared repeatedly as the top 5% of outliers over all five iterations of the Mahalanobis distance calculations were then identified as candidates and set aside for monosaccharide composition analysis (Figure 3b). From this particular example (batch number 800), 10 outliers out of 370 leaf samples were selected for HPAEC analysis.

Using the criteria defined above, we analyzed 11 batches in this manner to serve as our pilot study. In summary, the pilot study consisted of 550 mutant lines (3590 leaf samples), resulting in a set of 235 leaf samples that were determined to be Mahalanobis outliers. A total of 145 of these outliers and 73 references (Mahalanobis distance references as well as more arbitrarily chosen samples) were analyzed for cell wall monosaccharide composition. The reference samples were used to define cell wall percentage monosaccharide variation in the population relative to their batch. Unlike *Arabidopsis*, the xylose detection is not a problem with rice samples since we know that mannose is not detected in rice leaves at that stage of development (Ronald et al, unpublished results). Across references from the 11 batches, the relative percent variability for major cell wall monosaccharides was found to be 3.3 ± 2.3% for Arabinose (Ara), 12.5 ± 8.5% for Galactose (Gal), 14.2 ± 9.2% for Glucose (Glc) and 4.8 ± 3.0% for Xylose (Xyl). Minor cell wall monosaccharides (Rhamnose [Rha], Fucose [Fuc], Glucuronic Acid [GlcA], and Galacturonic Acid [GalA]) were excluded from further analyses due to large variations. In order to determine significant changes in cell wall sugar composition, we calculated the relative percent monosaccharide differences outside 4 standard deviations (*μ *± 4*σ; *99.99% confidence interval) for each of the major sugars based on the references and used these values as monosaccharide outlier identification criteria. Of the 145 outliers analyzed by HPAEC, a total of 48 (33% validation rate) had a significant sugar composition difference (> 4*σ*) in one or more of the major cell wall monosaccharides (Additional file 2: Table S1). Significant variations range from a single sugar difference (e.g. 0373-3, 0826-7, 1533-4 and 1784-8) up to variations in 3 major monosaccharides (e.g. 0230-3, 0352-2, 1536-1, 2258-2). These variations encompass both deficiencies as well as relative abundance changes among the monosaccharides analyzed.

### Modeling monosaccharide composition from FT-NIR spectra

A major advantage in using FT-NIR is the ability to derive quantitative information by means of PLS modeling. This is done by correlating known biochemical values (e.g. monosaccharide composition) of a calibration set (e.g. cell wall mutants) with the respective FT-NIR spectra. However, with the exception of *Arabidopsis*, no extensive and readily available collection of well documented cell wall mutants exists that could be used to develop a calibration set of varying biochemical characteristics. Consequently, for our rice mutant population, we reasoned that the set of Mahalanobis outliers that have been already identified and characterized in the pilot study would constitute a robust calibration set. Because these outliers span multiple batches grown at different times, they provide a range of cell wall monosaccharide compositions. Additionally, modeling of monosaccharides can allow us to make targeted detections of specific cell wall changes, which is not possible by using the Mahalanobis distance approach alone.

**Figure 4:**
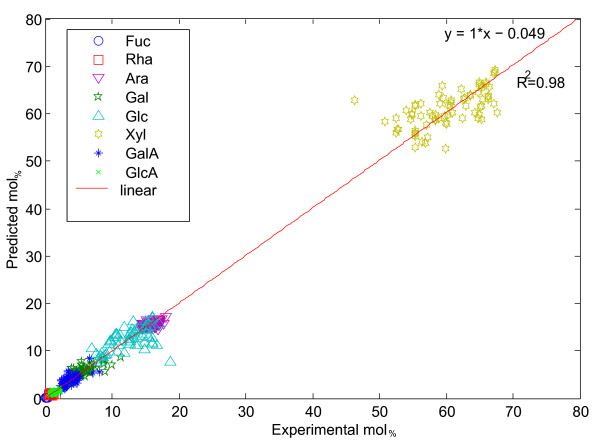
**Correlation analysis of PLS modeling using samples in calibration set**. A correlation analysis of predicted (PLS model of FT-NIR) versus experimentally determined monosaccharide composition (mol_%_) of rice leaf samples. Data represents the iterative process of *K*-fold cross validation of the 206 leaf samples analyzed by both FT-NIR and HPAEC. The correlation coefficient between predicted and experimental values was calculated to be *R^2 ^*= 0.98.

The Mahalanobis outliers and respective references varied by up to 3 mol_% _for Fuc, Rha, GlcA and GalA, while the more abundant sugars showed a larger variation spanning 5 mol_% _for Ara, 10 mol_% _for Glc and nearly 20 mol_% _percent for Xyl (Additional file 3: Figure S2). A majority of these samples (206) were selected as a calibration set for monosaccharide modeling of FT-NIR spectra. A model was constructed for each of the major sugars to correlate monosaccharide composition with FT-NIR spectra by PLS modeling. *K*-fold cross validation was used to assess the accuracy of the predictive model. This was done in an iterative manner by first, randomly dividing the calibration set into subsets (training and test set), second, constructing a model based on the training set, and finally validating the model with the test set. An analysis of predicted versus experimental mol_% _data for each sugar demonstrated the robustness of the calibration set in developing a predictive model with a correlation coefficient (*R^2^*) of 0.98 (Figure 4). This predictive test indicated that cell wall monosaccharide composition could be confidently predicted from FT-NIR spectra of an unprocessed rice leaf tissue.

Based on the PLS model constructed from the calibration set, the cell wall sugar composition for all the samples in the pilot set (550 lines) was predicted. The differences in predicted Ara, Xyl, Glc and Gal, representing the most abundant monosaccharides, were then used as new criteria for a re-analysis of outlier detection in the pilot study. For a given batch, the averages for the major sugars were determined based on predicted values for all the samples in the batch. Next, percent differences for an individual sample in the batch were calculated based on these batch averages and the predicted sugars for that particular sample. We employed the confidence interval threshold defined above (±4 *σ*; >99.99% confidence interval) for determining predicted significant sugar composition variants (Ara; ≥ Δ 9.1%, Gal; ≥ Δ 34.0%, Glc; ≥ Δ 36.6%, and Xyl; ≥Δ12.1%). Based on these criteria, 75 samples were predicted to have changes in cell wall composition and a randomly selected subset of 30 was analyzed for monosaccharide composition. A total of 18 samples were experimentally confirmed with significant sugar composition differences (Additional file 4: Table S2). This constitutes a 60% validation rate for the PLS model with regard to sugar composition. The model identified an additional seven outliers, bringing the total number to 55 total outliers out of a population of 3590 samples. This constitutes a rate of 1.3% outlier confirmation rate for the pilot study of 550 lines when both the Mahalanobis distance and model outliers are considered. Out of these 55, 11 were identified by both the Mahalanobis distance and the model.

**Figure 5:**
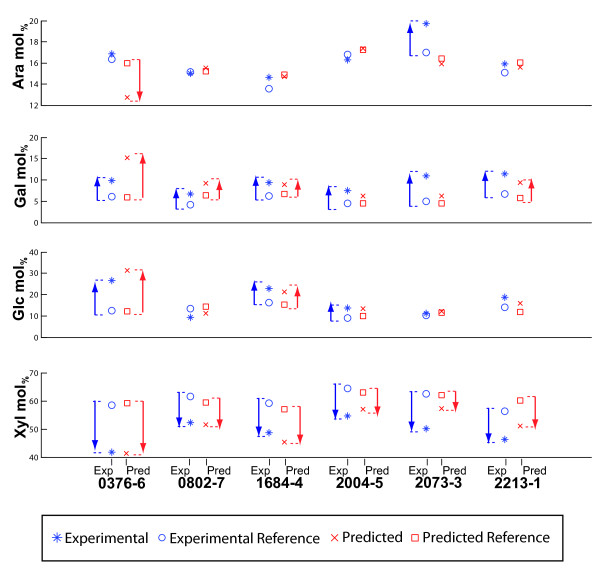
**Correlations of experimentally measured and predicted values of monosaccharides**. Diagrammatical representation of correlations between experimental and predicted major cell wall monosaccharides in an example set of six rice outliers. The similar trend of variation from the reference in both the predicted and experimental values demonstrates the precision of the PLS model. The experimental and predicted references (circle and square) indicate the average values (mol_%_) based on five non-outlier samples; the experimental values (asterisk) are values for individual samples (mol_%_); the predicted amounts (cross) are the values (mol_%_) determined by the model. The arrows indicate the deviation of the experimental or predicted value from the corresponding reference value, in mol_%_.

A set of 6 samples (outlined in Additional file 4: Table S2) was randomly selected to illustrate the predictive value of the model for identifying significant and inherent variations in sugar composition. The predicted and experimentally determined values for Ara, Gal, Glc, and Xyl for each of the six sample outliers are shown in Figure 5. All six outliers showed at least a 30% experimental Gal content variation compared to reference samples, and all had significant decreases in xylose content. For example, as can be seen in sample 0376-6, the model predicted a decrease in Xyl as well as increase in Gal and Glc contents, relative to the predicted reference values for all these sugars (Figure 5, red symbols). Biochemical analysis of this sample by HPAEC (experimental), confirmed the predicted changes in sugar composition for Gal, Glc and Xyl, but not as well for Ara (Figure 5, blue symbols). Overall, the majority of the predicted changes in sugar content were confirmed experimentally, with the highest success of prediction for changes in Gal and Xyl.

## Discussion

**Figure 6:**
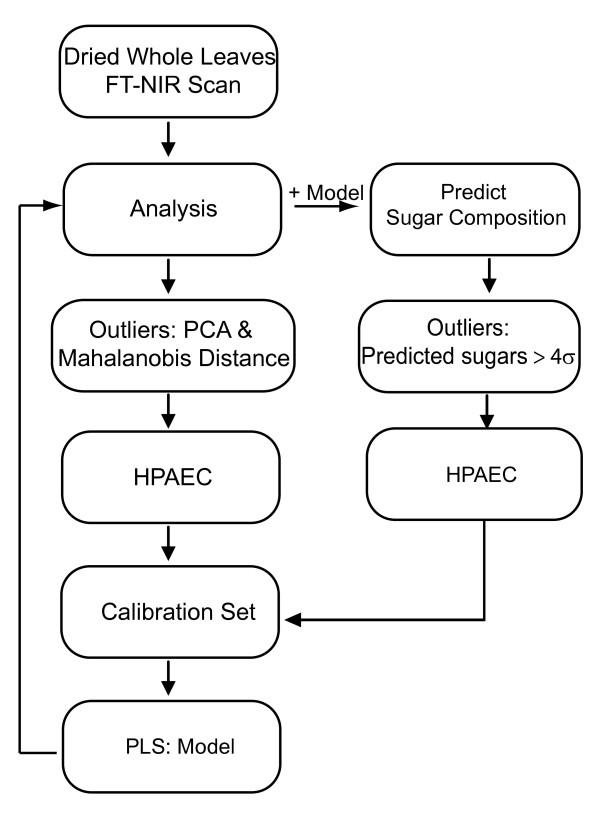
**Process schematic**. Outline of FT-NIR scanning and subsequent data processing involved in generating outliers followed by a calibration set for modeling. This illustrates the iterative process that can be implemented to improve outlier detection.

We have outlined a detailed application for FT-NIR in a plant cell wall composition screen that can be used in a non-destructive and rapid manner. We have shown that outlier identification performed by multivariate analysis of FT-NIR spectra using PCA and Mahalanobis distance has approximately a 30% validation rate for monosaccharide composition. Additionally, we have taken advantage of the quantitative nature of NIR to develop a process to derive a calibration set based on Mahalanobis distance outliers to create a model to predict monosaccharide composition from FT-NIR spectra alone. By incorporating PLS modeling into the screening methodology, the outlier detection rate was significantly improved to 60% compared to the Mahalanobis distance approach. These processes can be applied to any large population of plant samples without the need for a known or previously characterized collection of variants by following a multistep process (outlined in Figure 6). This allows for experimental validation on a subset of carefully selected candidates in a mutant or natural variant population, greatly increasing throughput and efficiency.

### Outlier detection by Mahalanobis distance

While data acquisition is straight forward, the quantitative examination of the FT-NIR spectra can only be achieved using robust multivariate analysis such as PCA and Mahalanobis distance. In previous studies with FT-MIR microspectroscopy, the Mahalanobis distance was successfully applied to the identification of putative cell wall mutants in flax [8] and in *Arabidopsis *[9]. Although a recent report briefly mentions the use of FT-NIR and Mahalanobis distance for the identification of maize cell wall mutants [30], neither validation nor a detailed analysis on the application of the method is provided. Although PCA by itself can be used to show clustering or outlier identification [30], this can only be done when the majority of the variances (90% or more) can be accounted for by the first few principal component scores, and if prior knowledge is available to determine which scores represent the variation of interest. In our study, we were unable to see any discrete clustering of mutants by plotting the first 2 principal component scores (e.g. *Arabidopsis *cell wall mutants Additional file 5: Figure S3). This was most likely due to the large biological variation between samples where more than 10 principal component scores were required to account for 90% of the variation. Consequently, in our study, PCA was only used as a data compression technique and required a linear discriminate technique (Mahalanobis distance) to serve as a metric to determine outlier classification from a reference. A requirement for using the Mahalanobis distance after PCA is that a reference set is needed to account for biological and technical variability to identify outliers from a defined population.

The applicability of the FT-NIR and Mahalanobis distance approach to identity cell wall composition differences was initially demonstrated using known cell wall mutants in both *Arabidopsis *and rice. While this initial study showed the validity of the approach, application of this technique is problematic when faced with biological variation prevalent in large-scale screening and with no defined reference set. Because plants grown at different times have been exposed to differing growth conditions, these would likely be reflected in the structure and composition of the cell wall. Therefore each batch in the pilot study using the rice population was treated separately and reference sets specific to the batch were required to account for variability unique to that batch.

Using this approach, we demonstrated that it is possible to identify outliers with one FT-NIR scan. Not all outliers identified by the Mahalanobis distance analysis proved to show significant monosaccharide composition differences discernible by HPAEC analysis of TFA hydrolysates. These most likely represent outliers for different reasons, for example changes in lignin, starch, cellulose content or could constitute developmental stage differences. This has been shown in previous studies that utilize Mahalanobis distance as the outlier detecting scheme. In a forward-genetic screen of maize mutants, 33 out of the 39 NIR Mahalanobis distance outliers showed no differences in cell wall composition and were identified as nir mutants with "invisible" phenotypes [30]. This was highlighted in our study of the *Arabidopsis *cell wall mutant analysis where *irx10-L*, a known xylan biosynthesis mutant that fails to show a clear morphological or sugar phenotype [35,36], but was an FT-NIR outlier in the Mahalanobis analysis. Similarly, the *Arabidopsis *cellulose deficient mutant *rsw1 *[34] and the rice *brittle culm *mutant (deficient in secondary cell wall cellulose deposition) were also found as outliers in the Mahalanobis distance analysis without showing changes in matrix polysaccharide sugar composition. It is known that *rsw1 *is a temperature-conditional mutant [34]. We grew *rsw1 *at the non-permissible temperature and still showed that it could be identified as an outlier compared to WT Col-0. We measured cellulose content in *rsw1*, *irx10-L *and *irx14 *and we could not find significant differences between wild type Col-0 and these mutants (Additional file 6: Figure S4). This underscores the possibility that additional changes, not previously reported in *rsw1*, account for the spectral differences shown here. While the correlation of FT-NIR with monosaccharide composition in this study only provides an insight into matrix polysaccharides, this approach could easily be broadened by correlating other cell wall components with FT-NIR spectra [38]. Therefore, other robust biochemical methods that can probe the content of other components in the sample could help to account for a proportion of other outliers we identified. It is then clear that FT-NIR can identify a range of changes in biological samples, in addition to variations in cell wall composition. In support of this hypothesis, a combination of FT-NIR and GC-TOF/MS profiling was recently applied to identify *Arabidopsis *mutants with changes in seed metabolite fingerprints [39].

### Predictive modeling of sugar composition

By incorporating predictive modeling of monosaccharide composition in a mutant screen, a more targeted outlier detection scheme can be implemented; however this can only be achieved after a robust calibration set is obtained. To generate calibration sets that encompass large variability for modeling, previous applications for biomass characteristics using NIR have used various plant tissue types [23,40]. That approach to modeling is not feasible in a mutant screen dealing with variability associated with a single tissue type. In order to derive a calibration set spanning a varied range of cell wall sugar compositions, we used the set of outliers determined by Mahalanobis distance analysis of the rice mutant population. We constructed a PLS model from the calibration set to correlate FT-NIR spectra with sugar composition, which allowed the prediction of sugar composition of every leaf sample (3590) that was scanned by FT-NIR. The power of this technique in a large population screen is that the model can be continuously improved upon as more candidates are identified and added to the calibration set. We chose to scan a rice leaf only once and therefore only a single scan is correlated to experimental monosaccharide composition. This initial step could be improved by undertaking multiple scans at various locations on the leaf that could improve both the Mahalanobis and PLS model outlier validation rate but will be time-consuming. In addition, other improvements in the model can be made by how the spectra is preprocessed [41]. The modeling process is limited by the quality of the biochemical method, thus if monosaccharide composition analysis by HPAEC contains inaccuracies, the prediction model error will also increase. For these reasons, low abundant sugars in the cell walls of rice samples such have Fuc, Rha, GalA and GlcA had a higher degree of associated prediction error. Consequently, for this study we focused our comparative analyses on Xyl, Ara, Gal and Glc.

Even though not all of the predicted Xyl, Ara, Glu and Gal differences correlated with experimental values for some of the mutants we discovered, at least one of the predicted monosaccharide changes from each sample would identify it as an outlier for further analysis. This is evident with mutant 2073-3 which was set aside as Gal outlier based on sugar prediction, but experimental monosaccharide composition analysis showed it to have significant changes in other sugars as well (Figure 5 and Additional file 4: Table S2). Once measured and confirmed, these samples would then serve as additional data points in the calibration set, and an improved model can be developed.

**Figure 7:**
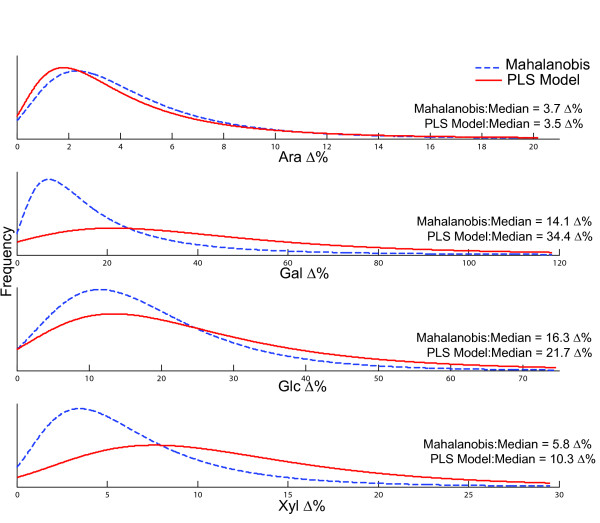
**Comparison of population distribution**. Normalized population distributions of major cell wall sugars from the experimentally determined Mahalanobis distance and PLS model outliers. The PLS model outliers display a shifted distribution for increased sugar differences for Gal, Gla and Xyl. PLS modeling of cell wall sugar composition provides a smaller and more selective cohort of possible outliers enriched in monosaccharide differences compared to using Mahalanobis distance alone.

Confirmed outliers were detected using both the Mahalanobis distance as well as PLS modeling but with different rates of validation. Out of the total analyzed rice leaf samples, 33% of the Mahalanobis outliers were confirmed to have large variation in the major cell wall monosaccharides versus 60% validation rate for the smaller population of PLS model outliers. The largest variations in Ara (0230-3), Glc (0376-6) and Xyl (0352-4) were found using both Mahalanobis and PLS modeling, with the exception of the largest Gal variant (2073-3) which was predicted only by the PLS model. In an analysis of the population distribution of all Mahalanobis distance and PLS model outliers versus the percent sugar difference, we can see a shift towards larger sugar variation for the PLS model outlier population than for the Mahalanobis population (Figure 7). This is most dramatic for Glc and Xyl showing almost a 2-fold increase in the median for the PLS model compared to Mahalanobis distance. Outlier confirmation for Ara, however, showed no significant improvement by PLS modeling compared to the Mahalanobis distance (Figure 7). In addition to improving the detection of confirmed sugar composition outliers, the model allows for the selection of defined sets of candidates for experimental validation, reducing the amount of samples to process biochemically. This model can potentially be used on plant materials that have the same range of monosaccharide composition as suggested by Liu et al [40]. Therefore a large mutant population can serve two purposes: one of providing a set of potential mutants, and the other as source of information that can be exploited to create a broad base model using NIR.

## Conclusion

This study has demonstrated a robust high-throughput application for FT-NIR on a single tissue type to identify cell wall composition changes. This approach is applicable to large-scale mutant and population analyses, as it requires minimal sample handling and additional well-established methods for data processing. The procedure can be done in a two-step process by first identifying outliers by Mahalanobis distance analysis, followed by a more targeted screen using a PLS model for monosaccharide composition. Additionally, this procedure can be continually improved upon during the screening process when more candidates are identified and confirmed. We identified 55 confirmed outliers with significant cell wall monosaccharide composition changes in this pilot study using a subset of a rice mutant population. After screening the entire population by Mahalanobis distance analysis or by sugar modeling of NIR spectra, all candidates will need to be validated by detailed segregation analyses in subsequent segregating generations.

## Methods

### Fourier Transform Near-Infrared Spectroscopy

A MPA FT-NIR Spectrometer (Bruker Optics) was used to measure samples. Spectral absorbance covering a range from 38000 to 12000 cm^-1 ^was taken at a spectral resolution of 8 cm^-1^. Spectra were collected in diffuse reflectance mode. A total of 32 scans were taken and co-added for each sample (10 seconds). Whole and dried *Arabidopsis *rosettes and rice leaves were placed directly on the sampling window for measurements.

### Data Preprocessing

Preprocessing of absorption spectra was done using Opus software (Bruker Optics). Absorption spectra were first cropped to 3800 cm^-1 ^to 9000 cm^-1^, smoothed using 25 points then baseline corrected.

### Statistical analysis and modeling

Statistical analysis was undertaken using the Statistical toolbox in Matlab (Mathworks). After pre-processing of the spectra, the data set was area-normalized then mean centered. Principal component analysis was used for data compression [42]. Mahalanobis distance is expressed as ${\left({\text{d}}_{ij}\right)}^{\text{2}}={\left({\bar{\text{u}}}_{i}-{\bar{\text{u}}}_{j}\right)}^{\text{T}}S^{-\text{1}}\left({\bar{\text{u}}}_{i}-{\bar{\text{u}}}_{j}\right)$ where ${\bar{\text{u}}}_{i}$ and ${\bar{\text{u}}}_{j}$ are the group means for 2 groups and **S **is the covariance matrix. A mathematical constraint in calculating the Mahalanobis distance forces that the number of variables (pc scores) cannot equal or exceed the number of observations (controls). Taking the inverse of a covariance matrix with fewer observations than the number of variables is not recommended therefore forcing the constraint that there cannot be more principal component scores (variables) than references (observations) when calculating the Mahalanobis distance. Calibration set: 12 samples were dropped from the 218 samples that were measured by HPAEC due to error in the FT-NIR spectra upon closer inspection. The remaining 206 files were subsequently used as the calibration set. PLS was performed using in-house programming using the plsregress function in matlab. Each monosaccharide was modeled individually with different number of fitting components to avoid overfitting of data (Arabinose: 8 components, Galactose: 7 components, Glucose: 11 components, Xylose: 10 components). A *K*-fold cross validation was used to validate our sugar prediction model and involves removing a randomly selected subset of data and assigning it as a test group then creating a model based on the remaining data. Values of the test group are then predicted and compared to real values [43]. This is repeated K times (K = 10 times) and a Root Mean Square Error of Prediction (RMSEP) was calculated for all 10 times and used as a metric to refine the partial least squares model and determine fitting parameters.

### Arabidopsis and rice growth conditions

*Arabidopsis *plants were grown in a growth chamber maintained at 22°C with 8 h photoperiod for 4 weeks after 2 days stratification at 4°C. Whole rosettes were harvested, sandwiched between filter paper (Whatman) and immediately placed in a 40°C oven to dry for 2 days. A total of 5 to 8 biological replicates were used for each analysis. Wild type plants were Col-0 or *qrt*, depending on the mutant background. Rice plants were grown as described in [44].

### Monosaccharide composition of cell wall material

Plant material (approximately 60 mg) was oven dried at 40°C and ground in a bead beater (Retsch) to a fine powder. Preparation and hydrolysis of alcohol-insoluble residues were prepared from five to eight replicates from *Arabidopsis *rosettes and individual rice leaves according to previous procedures [32]. Monosaccharide composition was measured by high-performance anion exchange chromatography with pulsed amperometric detection (HPAEC-PAD) (Dionex) using a CarboPac PA20 column using established procedures [32].

### Cellulose content determination

We used the Updegraff method for cellulose content estimation [45]. Briefly, samples are first hydrolyzed with acetic acid/nitric acid solution to remove matrix polysaccharides and amorphous cellulose. The remaining sample is digested with 67% sulfuric acid and glucose content is measured using the anthrone reagent method [46].

## Competing interests

The authors declare that they have no competing interests.

## Authors' contributions

ASM contributed in conceiving project, carried out multivariate analysis and drafted the manuscript. MC generated the rice mutant population and, along with WHST, collected and provided leaf samples. JL performed FT-NIR spectroscopy on rice samples and help perform TFA hydrolysis and HPAEC measurements. JLH contributed in manuscript preparation and part of the analysis of the modeling data. PCR/MEVS supervised experiments and preparation of manuscript and carried out HPAEC analysis. All authors have read and approved the final manuscript.

## Supplementary Material

Additional file 1 **Figure S1 Monosaccharide composition analysis of *Arabidopsis *cell wall mutants**. (a)HPAEC analysis of Col-0 (wt) and cell wall mutants (*irx10-L*, *irx14 *and *rsw1*). (b) HPAEC analysis of cell wall mutants *arad*1 and *xdgl-1 *compared to corresponding background *qrt*. Click here for file

Additional file 2 **Table S1 Monosaccharide composition of Mahalanobis distance rice outliers**. Samples from the rice mutant population with significant variation in one or more major cell wall monosaccharide identified by Mahalanobis distance and confirmed by biochemical analysis (HPAEC). Values are show as percentage variation from the reference values for each batch. Sugar changes greater than 4 standard deviations from the relative mean of reference samples (*μ *± 4*σ*) were employed to determine outliers. The sugars outside the 99.99% confidence interval for each major sugar are shown underlined with variations exceeding Ara (± 9.1%), Gal (± 34.0%), Glc (± 36.6%) or Xyl (± 12.1%) for any sugar. Click here for file

Additional file 3 **Figure S2 Monosaccharide composition range of rice samples identified by Mahalanobis distance**. Cell wall sugar composition of Mahalanobis sample outliers and references showing the range of sugar composition that were generated. This then served as the calibration set of the PLS model. Click here for file

Additional file 4 **Table S2 Monosaccharide composition range of rice samples identified by PLS modeling of NIR spectra**. Samples from the rice mutant population with significant variation in one or more major cell wall monosaccharide identified by the PLS model and confirmed by biochemical analysis (HPAEC). Values are show as percentage variation from the reference values for each batch. Sugar changes greater than 4 standard deviations from the relative mean of reference samples (*μ *± 4*σ*) were employed to determine outliers. The sugars outside the 99.99% confidence interval for each major sugar are shown underlined with variations exceeding Ara (± 9.1%), Gal (± 34.0%), Glc (± 36.6%) or Xyl (± 12.1%) for any sugar. Click here for file

Additional file 5 **Figure S3 Principal component analysis of *Arabidopsis *cell wall mutants**. PC1 versus PC2 plot on area-normalized and baseline corrected FT-NIR spectra of *Arabidopsis *cell wall mutants and corresponding background. There is no clear segregation between wildtype (Col-0) and mutants that would aid in the identification of outliers. Click here for file

Additional file 6 **Figure S4 Cellulose content of Arabidopsis cell wall mutants**. Cellulose content determined by Updegraff method. Click here for file

## References

[B1] Yuan JS Tiller KH Al-Ahmad H Stewart NR Stewart CN Jr Plants to power: bioenergy to fuel the future Trends Plant Sci 2008 13 421 429 10.1016/j.tplants.2008.06.001 18632303

[B2] Vega-Sánchez ME Ronald PC Genetic and biotechnological approaches for biofuel crop improvement Curr Opin Biotechnol 2010 21 218 224 10.1016/j.copbio.2010.02.002 20181473

[B3] Reiter WD Chapple C Somerville CR Mutants of *Arabidopsis thaliana *with altered cell wall polysaccharide composition Plant J 1997 12 335 345 10.1046/j.1365-313X.1997.12020335.x 9301086

[B4] Turner SR Somerville CR Collapsed xylem phenotype of Arabidopsis identifies mutants deficient in cellulose deposition in the secondary cell wall Plant Cell 1997 9 689 701 9165747 10.1105/tpc.9.5.689 PMC156949

[B5] Turner SR Taylor N Jones L Mutations of the secondary cell wall Plant Mol Biol 2001 47 209 219 10.1023/A:1010695818416 11554473

[B6] Gille S Hansel U Ziemann M Pauly M Identification of plant cell wall mutants by means of a forward chemical genetic approach using hydrolases Proc Natl Acad Sci USA 2009 106 14699 14704 10.1073/pnas.0905434106 19667208 PMC2731844

[B7] Chen LM Carpita NC Reiter WD Wilson RH Jeffries C McCann MC A rapid method to screen for cell-wall mutants using discriminant analysis of Fourier transform infrared spectra Plant J 1998 16 385 392 10.1046/j.1365-313x.1998.00301.x 9881159

[B8] Mouille G Robin S Lecomte M Pagant S Hofte H Classification and identification of Arabidopsis cell wall mutants using Fourier-Transform InfraRed (FT-IR) microspectroscopy Plant J 2003 35 393 404 10.1046/j.1365-313X.2003.01807.x 12887590

[B9] Vogel J Unique aspects of the grass cell wall Curr Opin Plant Biol 2008 11 301 307 10.1016/j.pbi.2008.03.002 18434239

[B10] Sene CFB McCann MC Wilson RH Grinter R Fourier-transform raman and fourier-transform infrared-spectroscopy - an investigation of 5 higher-plant cell-walls and their components Plant Physiol 1994 106 1623 1631 12232436 10.1104/pp.106.4.1623 PMC159706

[B11] McCann MC Defernez M Urbanowicz BR Tewari JC Langewisch T Olek A Wells B Wilson RH Carpita NC Neural network analyses of infrared spectra for classifying cell wall architectures Plant Physiol 2007 143 1314 1326 10.1104/pp.106.093054 17220361 PMC1820913

[B12] Dokken KM Davis LC Marinkovic NS Use of infrared microspectroscopy in plant growth and development Appl Spectrosc Rev 2005 40 301 326 10.1080/05704920500230898

[B13] Kacurakova M Wellner N Ebringerova A Hromadkova Z Wilson RH Belton PS Characterisation of xylan-type polysaccharides and associated cell wall components by FT-IR and FT-Raman spectroscopies Food Hydrocolloid 1999 13 35 41 10.1016/S0268-005X(98)00067-8

[B14] Kacurakova M Capek P Sasinkova V Wellner N Ebringerova A FT-IR study of plant cell wall model compounds: pectic polysaccharides and hemicelluloses Carbohydr Polym 2000 43 195 203 10.1016/S0144-8617(00)00151-X

[B15] Alonso-Simon A Encina AE Garcia-Angulo P Alvarez JM Acebes JL FTIR spectroscopy monitoring of cell wall modifications during the habituation of bean (*Phaseolus vulgaris *L.) callus cultures to dichlobenil Plant Sci 2004 167 1273 1281 10.1016/j.plantsci.2004.06.025

[B16] Robin S Lecomte M Hofte H Mouille G A procedure for the clustering of cell wall mutants in the model plant Arabidopsis based on Fourier-transform infrared (FT-IR) spectroscopy J Appl Stat 2003 30 669 681 10.1080/0266476032000053745

[B17] Coates JP The interpretation of infrared spectra: Published reference sources Appl Spectrosc Rev 1996 31 179 192 10.1080/05704929608000568

[B18] Watson CA Near IR reflectance spectrophotometric analysis of agricultural products Anal Chem 1977 49 A835 10.1021/ac50017a002

[B19] Wetzel DL Near-infrared reflectance analysis - sleeper among spectroscopic techniques Anal Chem 1983 55 1165 10.1021/ac00258a042

[B20] Norris KH Williams PC Optimization of mathematical treatments of raw near-infrared signal in the measurement of protein in hard red spring wheat.I. Influence of particle-size Cereal Chem 1984 61 158 165

[B21] Yeh TF Yamada T Capanema E Chang HM Chiang V Kadla JF Rapid screening of wood chemical component variations using transmittance near-infrared spectroscopy J Agric Food Chem 2005 53 3328 3332 10.1021/jf0480647 15853367

[B22] Tsuchikawa S A review of recent near infrared research for wood and paper Appl Spectrosc Rev 2007 42 43 71 10.1080/05704920601036707

[B23] Ye XP Liu L Hayes D Womac A Hong KL Sokhansanj S Fast classification and compositional analysis of cornstover fractions using Fourier transform near-infrared techniques Bioresour Technol 2008 99 7323 7332 10.1016/j.biortech.2007.12.063 18249535

[B24] Sanderson MA Agblevor F Collins M Johnson DK Compositional analysis of biomass feedstocks by near infrared reflectance spectroscopy Biomass Bioenerg 1996 11 365 370 10.1016/S0961-9534(96)00039-6

[B25] Gierlinger N Schwanninger M Hinterstoisser B Wimmer R Rapid determination of heartwood extractives in Larix sp by means of Fourier transform near infrared spectroscopy J Near Infrared Spectrosc 2002 10 203 214 10.1255/jnirs.336

[B26] Jin SY Chen HZ Near-infrared analysis of the chemical composition of rice straw Ind Crop Prod 2007 26 207 211 10.1016/j.indcrop.2007.03.004

[B27] Nkansah K Dawson-Andoh B Rapid characterization of biomass using fluorescence spectroscopy coupled with multivariate data analysis. I. Yellow poplar (Liriodendron tulipifera L.) J Renew Sustain Energy 2010 2 12 10.1016/j.biortech.2009.12.046 20163955

[B28] Geladi P Kowalski BR Partial least-squares regression: a tutorial Anal Chim Acta 1986 185 1 17 10.1016/0003-2670(86)80028-9

[B29] Vermerris W Saballos A Ejeta G Mosier NS Ladisch MR Carpita NC Molecular breeding to enhance ethanol production from corn and sorghum stover Crop Sci 2007 47 S142 S153

[B30] Penning BW Hunter CT Tayengwa R Eveland AL Dugard CK Olek AT Vermerris W Koch KE McCarty DR Davis MF Genetic resources for maize cell wall biology Plant Physiol 2009 151 1703 1728 10.1104/pp.109.136804 19926802 PMC2785990

[B31] Jensen JK Sorensen SO Harholt J Geshi N Sakuragi Y Moller I Zandleven J Bernal AJ Jensen NB Sorensen C Identification of a xylogalacturonan xylosyltransferase involved in pectin biosynthesis in Arabidopsis Plant Cell 2008 20 1289 1302 10.1105/tpc.107.050906 18460606 PMC2438468

[B32] Harholt J Jensen JK Sorensen SO Orfila C Pauly M Scheller HV ARABINAN DEFICIENT 1 is a putative arabinosyltransferase involved in biosynthesis of Pectic Arabinan in Arabidopsis Plant Physiol 2006 140 49 58 16377743 10.1104/pp.105.072744 PMC1326030

[B33] Brown DM Goubet F Vicky WWA Goodacre R Stephens E Dupree P Turner SR Comparison of five xylan synthesis mutants reveals new insight into the mechanisms of xylan synthesis Plant J 2007 52 1154 1168 10.1111/j.1365-313X.2007.03307.x 17944810

[B34] Peng L Hocart CH Redmond JW Williamson RE Fractionation of carbohydrates in Arabidopsis root cell walls shows that three radial swelling loci are specifically involved in cellulose production Planta 2000 211 406 414 10.1007/s004250000301 10987560

[B35] Brown DM Zhang ZN Stephens E Dupree P Turner SR Characterization of IRX10 and IRX10-like reveals an essential role in glucuronoxylan biosynthesis in Arabidopsis Plant J 2009 57 732 746 10.1111/j.1365-313X.2008.03729.x 18980662

[B36] Wu AM Rihouey C Seveno M Hornblad E Singh SK Matsunaga T Ishii T Lerouge P Marchant A The Arabidopsis IRX10 and IRX10-LIKE glycosyltransferases are critical for glucuronoxylan biosynthesis during secondary cell wall formation Plant J 2009 57 718 731 10.1111/j.1365-313X.2008.03724.x 18980649

[B37] Tanaka K Murata K Yamazaki M Onosato K Miyao A Hirochika H Three distinct rice cellulose synthase catalytic subunit genes required for cellulose synthesis in the secondary wall Plant Physiol 2003 133 73 83 10.1104/pp.103.022442 12970476 PMC196581

[B38] Bailleres H Davrieus F Pichavant FH Near infrared analysis as a tool for rapid screening of some major wood characteristics in a eucalyptus breeding program Ann For Sci 2002 59 479 490 10.1051/forest:2002032

[B39] Suzuki M Kusano M Takahashi H Nakamura Y Hayashi N Kobayashi M Ichikawa T Matsui M Hirochika H Saito K Rice-Arabidopsis FOX line screening with FT-NIR-based fingerprinting for GC-TOF/MS-based profiling Metabolomics 2010 6 137 145 10.1007/s11306-009-0182-2

[B40] Liu L Ye XP Womac AR Sokhansanj S Variability of biomass chemical composition and rapid analysis using FT-NIR techniques Carbohydr Polym 2010 81 820 829 10.1016/j.carbpol.2010.03.058

[B41] Andersen CM Bro R Variable selection in regression-a tutorial J Chemometr 2010 24 728 737 10.1002/cem.1360

[B42] Krzanowski WJ Principles of multivariate analysis: a user's perspective 2000 Revised Oxford: Oxford University Press

[B43] Stone M Cross-validatory choice and assessment of statistical predictions J R Stat Soc Ser B-Methodol 1974 36 111 147

[B44] Bart RS Chern M Vega-Sanchez ME Canlas P Ronald PC Rice Snl6, a cinnamoyl-CoA reductase-like gene family member, is required for NH1-mediated immunity to Xanthomonas oryzae pv. oryzae PLoS Genet 2010 6 10.1371/journal.pgen.1001123 PMC2940737 20862311

[B45] Updegraf.Dm Semimicro Determination of Cellulose in Biological Materials Anal Biochem 1969 32 420 10.1016/S0003-2697(69)80009-6 5361396

[B46] Scott TA Melvin EH Determination of dextran with anthrone Anal Chem 1953 25 1656 1661 10.1021/ac60083a023

