# Supplementary figures and images for: Combining multivariate analysis and monosaccharide composition modeling to identify plant cell wall variations by Fourier Transform Near Infrared spectroscopy
Source: Plant Methods. 2011 Aug 18;7:26. doi: 10.1186/1746-4811-7-26 (PMC3168417; doi:10.1186/1746-4811-7-26)

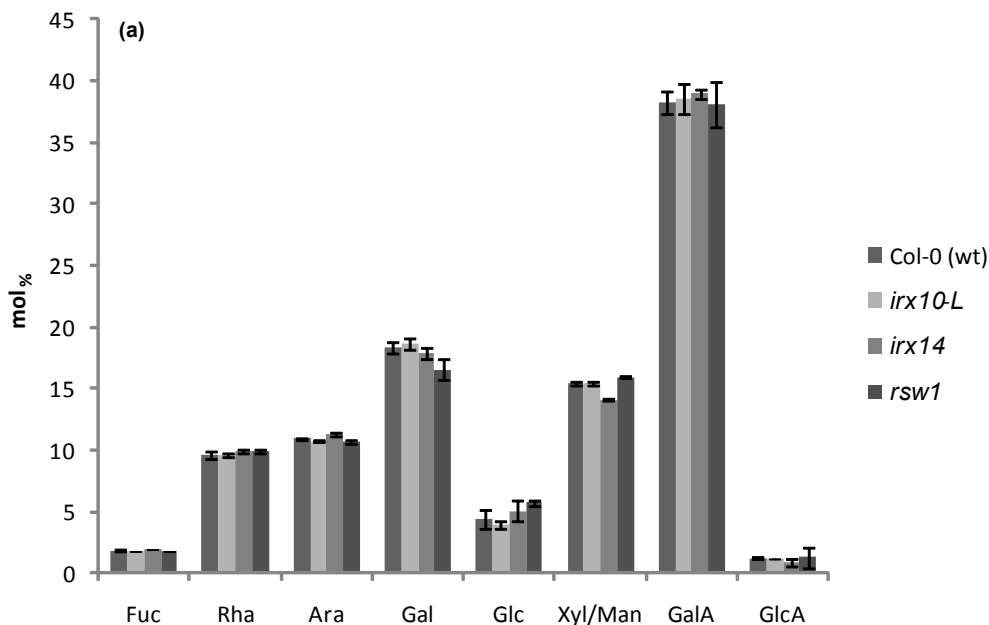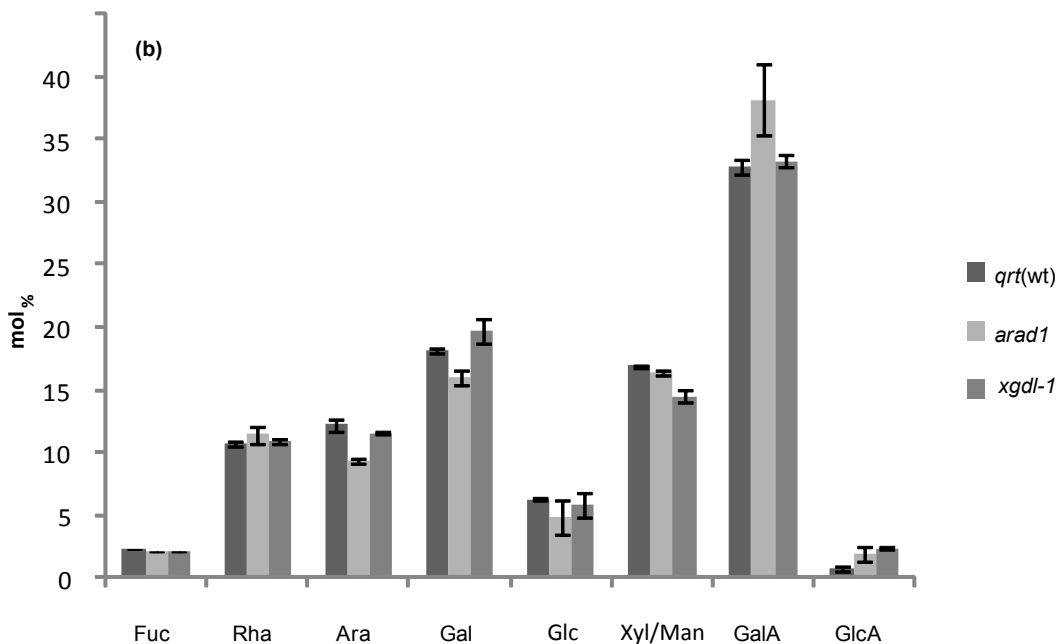

Supplement: Additional file 1 — Figure S1 Monosaccharide composition analysis of Arabidopsis cell wall mutants. (a)HPAEC analysis of Col-0 (wt) and cell wall mutants (irx10-L, irx14 and rsw1). (b) HPAEC analysis of cell wall mutants arad1 and xdgl-1 compared to corresponding background qrt. [file 1746-4811-7-26-S1.PDF]

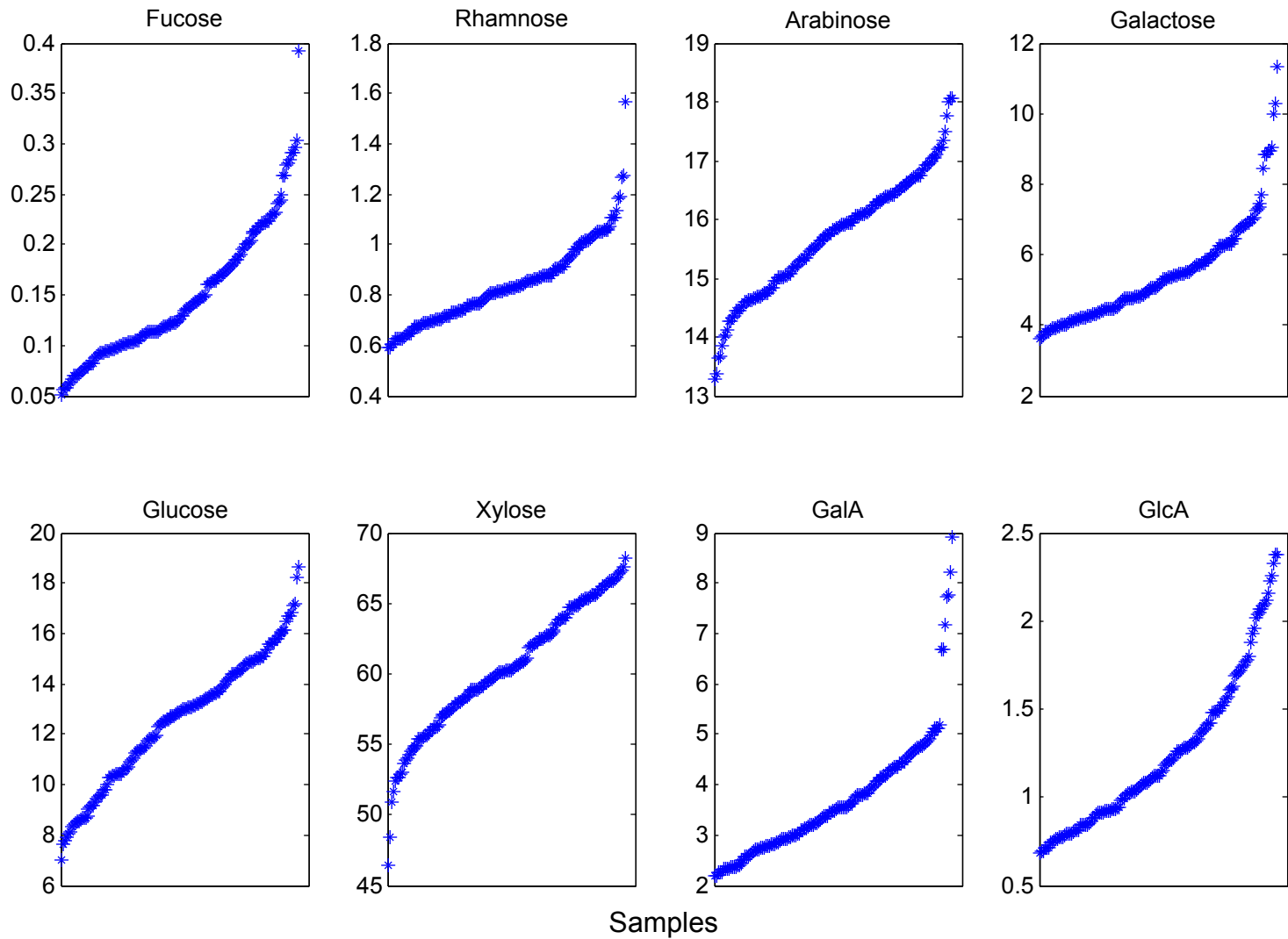

Supplement: Additional file 3 — Figure S2 Monosaccharide composition range of rice samples identified by Mahalanobis distance. Cell wall sugar composition of Mahalanobis sample outliers and references showing the range of sugar composition that were generated. This then served as the calibration set of the PLS model. [file 1746-4811-7-26-S3.PDF]

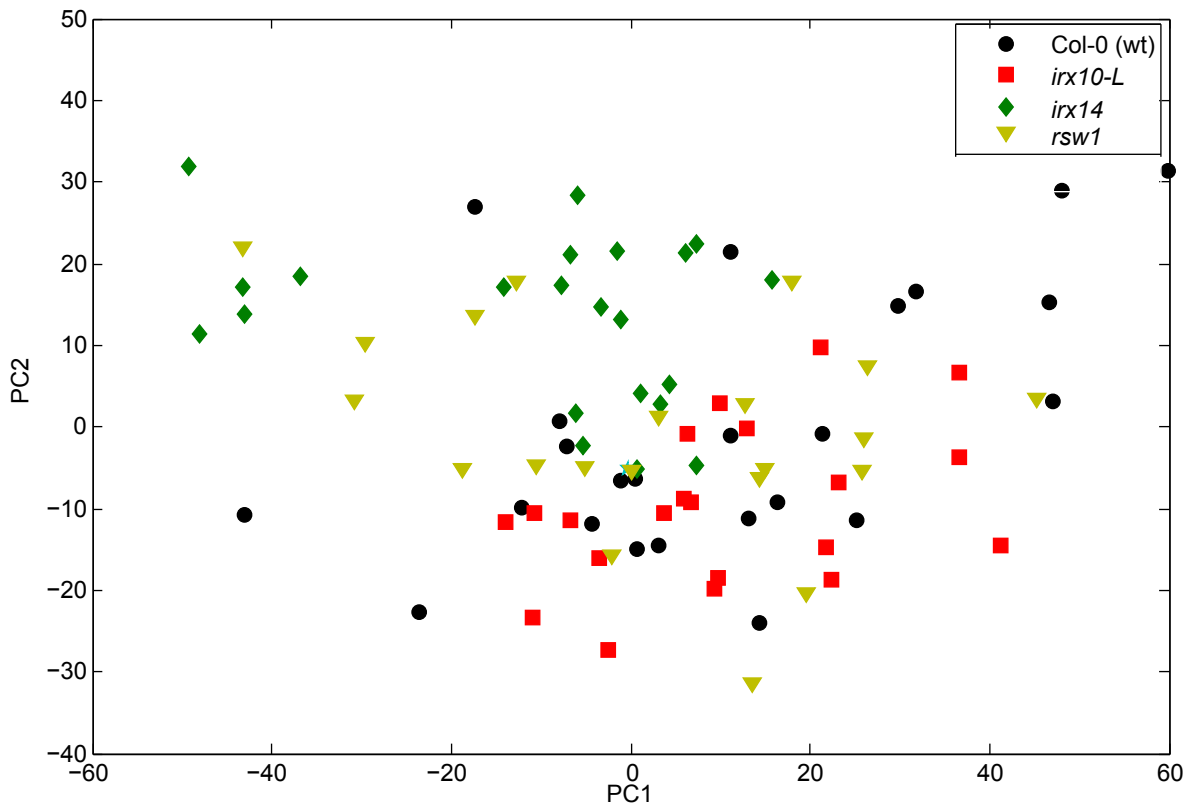

Supplement: Additional file 5 — Figure S3 Principal component analysis of Arabidopsis cell wall mutants. PC1 versus PC2 plot on area-normalized and baseline corrected FT-NIR spectra of Arabidopsis cell wall mutants and corresponding background. There is no clear segregation between wildtype (Col-0) and mutants that would aid in the identification of outliers. [file 1746-4811-7-26-S5.PDF]

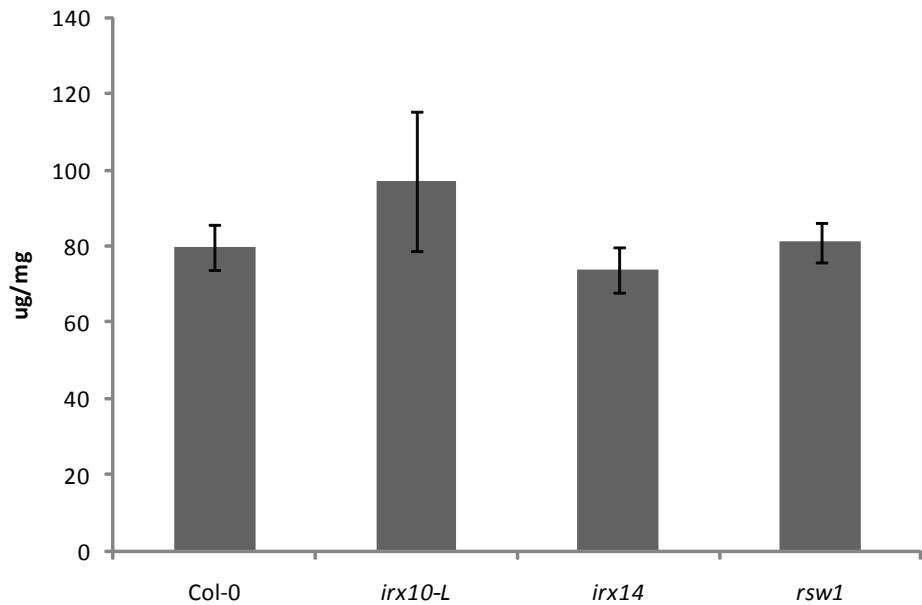

Supplement: Additional file 6 — Figure S4 Cellulose content of Arabidopsis cell wall mutants. Cellulose content determined by Updegraff method. [file 1746-4811-7-26-S6.PDF]
