# Supplementary material for: Combining multivariate analysis and monosaccharide composition modeling to identify plant cell wall variations by Fourier Transform Near Infrared spectroscopy
Source: Plant Methods. 2011 Aug 18;7:26. doi: 10.1186/1746-4811-7-26 (PMC3168417; doi:10.1186/1746-4811-7-26)
Supplement: Additional file 2 — Table S1 Monosaccharide composition of Mahalanobis distance rice outliers. Samples from the rice mutant population with significant variation in one or more major cell wall monosaccharide identified by Mahalanobis distance and confirmed by biochemical analysis (HPAEC). Values are show as percentage variation from the reference values for each batch. Sugar changes greater than 4 standard deviations from the relative mean of reference samples (μ ± 4σ) were employed to determine outliers. The sugars outside the 99.99% confidence interval for each major sugar are shown underlined with variations exceeding Ara (± 9.1%), Gal (± 34.0%), Glc (± 36.6%) or Xyl (± 12.1%) for any sugar. [file 1746-4811-7-26-S2.PDF]

Sample	Batch	Ara Δ%	Gal Δ%	Glc Δ%	Xyl Δ%
0202-2	1	-10.2%	-11.8%	-0.6%	2.9%
0209-4	1	9.1%	49.5%	-23.2%	-12.8%
0230-3	1	20.6%	37.7%	-13.8%	-15.8%
0237-6	1	7.0%	47.5%	13.3%	-17.0%
0244-4	1	9.9%	-10.0%	-30.8%	0.8%
0244-6	1	10.4%	-10.4%	-38.3%	2.5%
0352-2	2	-4.6%	101.1%	63.6%	-28.6%
0352-4	2	-1.2%	120.8%	63.3%	-30.1%
0352-6	2	13.8%	24.6%	-30.8%	0.7%
0352-7	2	1.5%	37.9%	-1.6%	-1.9%
0357-3	2	6.4%	57.4%	23.9%	-11.8%
0373-3	2	11.4%	18.9%	-34.2%	4.7%
0376-6	2	3.7%	81.4%	76.1%	-29.5%
0378-3	2	14.6%	69.9%	-4.4%	-7.7%
0383-6	2	6.1%	52.2%	3.3%	-7.3%
0512-3	3	9.3%	33.7%	2.8%	-9.2%
0802-7	4	-0.7%	59.9%	-30.1%	-14.9%
0808-6	4	1.3%	65.4%	-4.3%	-5.9%
0826-7	4	1.7%	103.1%	1.9%	-11.3%
0847-5	4	-2.7%	46.1%	11.3%	-6.6%
1502-3	5	-10.6%	5.0%	-20.2%	9.1%
1528-6	5	3.2%	-12.5%	-46.4%	12.5%
1533-2	5	4.0%	32.2%	23.8%	-12.4%
1533-4	5	1.9%	-5.7%	-40.4%	9.9%
1536-1	5	1.0%	41.9%	39.9%	-13.9%
1653-4	6	0.5%	46.5%	-9.1%	-5.4%
1663-1	6	9.2%	67.0%	-10.9%	-8.4%
1663-2	6	8.7%	45.3%	5.7%	-11.1%
1663-4	6	-2.0%	-22.2%	-29.1%	12.5%
1684-1	6	7.8%	43.1%	-18.7%	-3.9%
1684-4	6	7.8%	51.5%	40.1%	-18.0%
1689-5	6	13.5%	-3.5%	7.4%	-3.1%
1691-2	6	5.1%	29.7%	36.6%	-12.8%
1761-1	7	6.9%	45.6%	-1.6%	-3.9%
1765-4	7	-9.6%	-7.7%	31.7%	-8.4%
1766-1	7	-12.8%	-12.7%	24.5%	-10.0%
1781-1	7	9.6%	-2.4%	-31.3%	8.9%
1784-8	7	7.2%	-21.8%	-32.9%	12.6%
2003-1	8	0.5%	52.8%	16.1%	-11.8%

2205-3	10	12.1%	-8.0%	-18.8%	3.2%
2213-1	10	5.2%	67.6%	34.2%	-17.7%
2213-3	10	14.0%	-1.7%	-15.6%	1.5%
2220-1	10	4.2%	47.5%	6.8%	-6.9%
2234-8	10	5.4%	-37.8%	-24.9%	14.1%
2258-2	11	12.3%	92.2%	30.0%	-19.6%
2266-5	11	-1.7%	35.6%	32.0%	-11.1%
2275-2	11	0.6%	49.8%	8.6%	-9.9%
2278-5	11	10.5%	57.1%	-5.0%	-8.6%
2281-1	11	2.1%	35.9%	10.0%	-6.2%
